# Development and clinical validation of a droplet digital PCR assay for detecting *Acinetobacter baumannii* and *Klebsiella pneumoniae* in patients with suspected bloodstream infections

**DOI:** 10.1002/mbo3.1247

**Published:** 2021-11-16

**Authors:** Yang Zheng, Jun Jin, Ziqiang Shao, Jingquan Liu, Run Zhang, Renhua Sun, Bangchuan Hu

**Affiliations:** ^1^ Intensive Care Unit Zhejiang Provincial People’s Hospital People’s Hospital of Hangzhou Medical College Hangzhou China

**Keywords:** *Acinetobacter baumannii*, bloodstream infection, droplet digital polymerase chain reaction, *Klebsiella pneumoniae*, pathogen

## Abstract

The relatively long turnaround time and low sensitivity of traditional blood culture‐based diagnosis may delay effective antibiotic therapy for patients with bloodstream infections (BSIs). A rapid and sensitive pathogen detection method is urgently required to reduce the morbidity and mortality associated with BSIs. *Acinetobacter baumannii* and *Klebsiella pneumoniae* are two major microorganisms that cause BSIs. Here we report a novel droplet digital polymerase chain reaction (ddPCR) assay that can detect *A*. *baumannii* and *K*. *pneumoniae* in blood samples within 4 h, with a specificity of 100% for each strain and a limit of detection at 0.93 copies/μl for *A*. *baumannii* and 0.27 copies/μl for *K*. *pneumoniae*. Clinical validation of 170 patients with suspected BSIs showed that compared to blood cultures that detected four (2.4%) *A*. *baumannii* cases and seven (4.1%) *K*. *pneumoniae* cases, ddPCR detected 23 (13.5%) *A*. *baumannii* cases, 26 (15.3%) *K*. *pneumoniae* cases, and four (2.4%) co‐infection cases, including the 11 cases detected via blood culture. In addition, patients who tested positive via ddPCR alone (*n* = 42) had significantly lower serum concentrations of procalcitonin and lactate, SOFA and APACHE II scores, and 28‐day mortality than those reported positive via both blood culture and ddPCR (*n* = 11), suggesting that patients with less severe symptoms can potentially benefit from ddPCR‐based diagnosis. In conclusion, our study suggests that ddPCR represents a sensitive and rapid method for identifying causal pathogens in blood samples and guiding treatment decisions in the early stages of BSIs.

## Graphical abstract

We found that a total of 23 *Acinetobacter baumannii*, 26 *Klebsiella pneumoniae*, and 4 co‐infection cases out of 170 blood samples from critically ill patients with highly suspected bloodstream infections were detected by ddPCR (positive detection rates: 13.5%, 15.3%, and 2.4%, respectively). In contrast, only 4 (2.4%, 4/170) *A. baumannii* and 7 (4.1%, 7/170) *K. pneumoniae* that were detected by ddPCR were positively cultured, leading to a relative sensitivity of 100% for ddPCR.

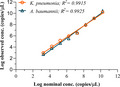

## 1 INTRODUCTION

Bloodstream infections (BSIs) represent a major cause of death worldwide, contributing to increases in healthcare costs, length of hospital stay, and in‐hospital morbidity (McNamara et al., 2018). Timely and accurate pathogen identification is critical to select the appropriate antimicrobial treatment for patients in the early stages of BSI. Blood culture remains the gold standard for identifying pathogens in BSIs (Blevins & Bronze, 2010). However, it is limited by its low sensitivity and long turnaround time (Riedel, & Carroll, 2016; Tabak et al., 2018). In patients with sepsis within the first 6 h of documented hypotension, every 1‐h delay in an appropriate antibiotic therapy leads to an average increase in the mortality rate of 7.6% (Kumar et al., 2006). For hospitalized patients with bacterial infections, inappropriate initial antimicrobial treatment almost doubles the risk of 30‐day mortality (Fraser et al., 2006). Thus, it is necessary to develop a rapid and accurate method for identifying causal pathogens in BSIs.

Culture‐independent, real‐time polymerase chain reaction (PCR)‐based, or microarray‐based methods, such as SeptiFast (Roche), Magicplex (Seegene), and TaqMan array card assay (Academy of Military Medical Science, Beijing, China), show promise in rapidly identifying pathogens and guiding early targeted antibiotic therapy in BSIs. However, their low sensitivities, ranging from 29% to 79.4%, may limit their clinical application (Buehler et al., 2016; Riedel, & Carroll, 2016; Warhurst et al., 2015; Zboromyrska et al., 2019; Zhang et al., 2018). Recently, droplet digital PCR (ddPCR) has been developed as a novel molecular diagnostic technique for the accurate detection and absolute quantitation of nucleic acids without the need of generating a calibration curve (Huggett et al., 2015). In ddPCR, the template is separated into thousands of nanolitre‐sized droplets and amplified. After amplification, the number of positive and negative reactions is counted, and the copy number of the template is calculated using Poisson statistics. So, ddPCR has been increasingly applied as a versatile tool with high sensitivity, accuracy, and precision in multiple clinical scenarios, including oncology (Postel et al., 2018), non‐invasive prenatal testing (Tan et al., 2019), and diagnosis of infectious diseases (Kelley et al., 2013; Pholwat et al., 2013; Sedlak et al., 2014; Sedlak, Cook, Huang, et al., 2014). Furthermore, few studies report that ddPCR is superior to conventional methods in its application for the detection of infectious pathogens, including bacteria, fungi, and viruses (Chen et al., 2021; Park et al., 2021; Wouters et al., 2020).

Circulating cell‐free (cf) DNA molecules originate from dying cells and from colonizing or invading microorganisms, which release DNA fragments into the blood as they break down, and their detection has emerged as a powerful non‐invasive diagnostic tool in the fields of prenatal screening, transplantation, and oncology (Aravanis et al., 2017; Bloom et al., 2017; Fan et al., 2008). An increasing number of studies have also indicated that microbial cfDNA detection may enable the reliable identification of various infections, such as invasive fungal infection, tuberculosis, and sepsis (Han et al., 2020). Grumaz et al. observed significantly increased levels of cfDNA derived from pathogenic bacteria in the plasma of patients with sepsis, and subsequently reported that microbial cfDNA‐based next‐generation sequencing (NGS) can provide higher sensitivity and specificity than that obtained via blood culture for the diagnosis of sepsis (Grumaz et al., 2016,2019). Moreover, in a case with confirmed *Acinetobacter baumannii* BSI, Liao et al. found that 1 ml of plasma contains more than 1000 copies of the *A*. *baumannii* genome, which exceeds the microbial load of intact live bacteria in the majority of blood samples derived from patients with sepsis (Liao et al., 2020). Thus, detection of microbial cfDNA may provide higher sensitivity for the diagnosis of BSIs than that of detecting intact live pathogens in blood samples. Additionally, in comparison with microbial cfDNA, the direct detection of pathogenic DNA in whole blood may be limited by the high quantity of human DNA that interferes with primer and probe binding in species‐specific PCR assays.

*A*. *baumannii* and *Klebsiella pneumoniae* represent two major Gram‐negative bacteria involved in BSIs and show a high capability to develop antibiotic resistance. BSIs caused by multidrug‐resistant *A*. *baumannii* and *K*. *pneumoniae* significantly contribute to mortality in intensive care units (ICUs), with a mortality rate of over 50% (Balkhair et al., 2019; Brink, 2019). In this study, we developed and validated a ddPCR‐based method to detect *A*. *baumannii* and *K*. *pneumoniae* in blood samples from patients with suspected BSI. Our results suggest that ddPCR represents a promising method for the accurate and rapid diagnosis of BSIs caused by *A*. *baumannii* and *K*. *pneumoniae*.

## 2 MATERIALS AND METHODS

### 2.1 Patients

A total of 170 patients were recruited from Zhejiang Provincial People's Hospital, Hangzhou, China, from March 2019 to October 2020. The inclusion criteria were age >18 years and suspected BSI. Suspected BSI was defined based on a sudden high fever (temperature ≥38.5°C) accompanied by hemodynamic instability that could not be explained by a site‐specific infection at another body site, and an increase of 2 points or more in the sepsis‐related organ failure assessment (SOFA) score as previously described (Hu et al., 2021). The demographic and clinical characteristics of each patient were collected within the first 24 h of suspicion of BSI; details are summarized in Table 1. Telephone follow‐up interviews were conducted with the surviving patients. An unfavorable outcome was defined as 28‐day all‐cause mortality after ICU admission.

**TABLE 1:** Comparison of clinical characteristics of patients tested positive by different detection methods

Clinical characteristics	Positive patients based on BC and/or ddPCR (*n* = 53)	ddPCR only (+) (*n* = 42)	BC & ddPCR (+) (*n* = 11)	*p*
Age (years)	65.7 ± 13.3	64.7 ± 13.8	69.4 ± 11.2	0.29
Male, *n* (%)	38 (71.7)	30 (71.4)	8 (72.7)	0.93
Use of vasoactive drugs	35 (66.0)	27 (64.3)	8 (72.7)	0.60
Norepinephrine, *n* (%)	34 (64.2)	26 (61.9)	8 (72.7)	0.51
Epinephrine, *n* (%)	6 (11.3)	5 (11.9)	1 (9.10)	0.79
Vasopressin, *n* (%)	12 (22.6)	8 (19.1)	4 (36.5)	0.22
Mechanical ventilation, *n* (%)	44 (83.2)	35 (83.3)	9 (81.8)	0.91
Renal replacement therapy, *n* (%)	14 (26.4)	11 (26.2)	3 (27.3)	0.94
Physical examinations				
Temperature (°C)	38.7 ± 0.37	38.8 ± 0.39	38.6 ± 0.21	0.10
Systolic blood pressure (mm Hg)	86.4 ± 17.6	88.4 ± 15.7	78.6 ± 22.8	0.11
Diastolic blood pressure (mm Hg)	44.2 ± 9.57	46.4 ± 8.10	41.5 ± 7.88	0.09
Complete blood counts and blood biochemistry				
Platelet counts, median (IQR) ×10^9^/L	61.2 (54.4–93.4)	83.0 (63.5–108.5)	40.3 (15.7–103.6)	0.12
White blood cell, median (IQR) ×10^9^/L	10.1 (7.72–13.3)	10.5 (8.19–13.3)	8.99 (3.02–26.7)	0.77
C‐reactive protein (mg/L), median (IQR)	152.1 (125.0–185.1)	140.6 (110.4–179.1)	204.4 (150.4–277.8)	0.06
Procalcitonin (pg/L), median (IQR)	4.84 (3.04–7.72)	3.70 (2.20–6.21)	12.9 (4.13–40.4)	0.03
Serum creatinine (μM), median (IQR)	125.6 (107.2–147.1)	119.2 (98.9–143.6)	151.5 (103.9–220.9)	0.22
Serum lactate (mM), median (IQR)	3.01 (2.50–3.62)	2.76 (2.28–3.43)	4.26 (2.75–6.89)	0.04
SOFA score	11.2 ± 4.77	10.5 ± 4.54	13.8 ± 4.85	0.03
APACHE II score	23.2 ± 7.46	21.9 ± 6.63	27.9 ± 8.77	0.02
28‐day mortality, *n* (%)	34 (64.2)	24 (70.6)	10 (90.1)	0.04

Note

Values are presented as mean ± standard deviation or number of subjects (percentage of the column total). The *p* values for characteristic differences were calculated for comparisons using the standard normal *z*‐test (mean) or Fisher's exact test (proportions).

Abbreviations: APACHE II, Acute Physiology and Chronic Health Evaluation II score; BC, blood culture; ddPCR, droplet digital PCR; IQR, interquartile range; SOFA, Sepsis‐related Organ Failure Assessment Score.

### 2.2 Blood culture and control bacterial strains

Upon preliminary confirmation of BSI, whole blood samples were obtained for blood culture and molecular diagnosis. Two sets of blood cultures were collected from each patient according to routine clinical practice; each set consisted of one aerobic bottle and one anaerobic bottle. The blood cultures were incubated at 37°C in a BacT/ALERT^®^ 3D System (BioMérieux). When a positive signal was obtained, Gram staining was performed, followed by subculturing on a Columbia blood agar plate at 37°C with 5% CO_2_. Following overnight incubation, the pathogens were further identified via matrix‐assisted laser desorption‐ionization time‐of‐flight mass spectrometry (VITEK^®^ MS system; BioMérieux). The positive control bacteria comprised *A*. *baumannii* ATCC 19606 along with five clinical isolates and *K*. *pneumoniae* CMCC 46117 along with 33 clinical isolates. A total of 131 other clinical isolates commonly found in BSIs were used as negative controls (Table 2).

**TABLE 2:** Microorganisms used in specificity assay

Organism	No.	Source/strain
*Acinetobacter baumannii*	5	ATCC 19606; Clinical isolates (4)
*Klebsiella pneumoniae*	33	CMCC 46117; Clinical isolates (32)
*Escherichia coli*	8	Clinical isolates (8)
*Pseudomonas aeruginosa*	19	CMCC 10104; Clinical isolates (18)
*Enterococcus faecalis*	9	ATCC 19433; Clinical isolates (8)
*Enterococcus faecium*	5	ATCC 19434; Clinical isolates (4)
*Serratia marcescens*	7	CMCC 41002; Clinical isolates (6)
*Salmonella enterica*	1	CMCC 41002
*Streptococcus pneumoniae*	1	CMCC 31001
*Enterobacter cloacae*	15	CMCC 43501; Clinical isolates (14)
*Burkholderia cepacia*	13	ATCC 25416; Clinical isolates (12)
*Enterobacter aerogenes*	1	ATCC 13048
*Proteus mirabilis*	18	BNCC 107943; Clinical isolates (17)
*Candida albicans*	2	CMCC 98001; Clinical isolates (1)
*Candida glabrata*	1	Clinical isolates (1)
*Stenotrophomonas maltophilia*	3	Clinical isolates (3)
*Staphylococcus aureus*	9	Clinical isolates (9)
*Staphylococcus epidermidis*	3	Clinical isolates (3)
*Staphylococcus hominis*	1	Clinical isolates (1)
*Staphylococcus haemolyticus*	13	Clinical isolates (13)
*Staphylococcus capitis*	2	Clinical isolates (2)

### 2.3 DNA extraction

For control bacterial strains, total DNA was isolated from 1 ml of overnight bacterial culture using a TIANamp bacteria DNA kit (TIANGEN Biotech) following the manufacturer's instructions. For blood samples, plasma was obtained via centrifugation at 1600 × *g* for 20 min. The DNA was extracted from 2 ml of plasma containing 1 μl of internal control using a magnetic serum/plasma DNA kit (TIANGEN Biotech) and an Auto‐Pure20B nucleic acid purification system (Hangzhou Allsheng Instruments). The DNA was eluted in 50 μl of elution buffer and stored at −80°C until use.

### 2.4 Primers and probes

Primers and TaqMan MGB probes (Table 3) were designed using Primer Express (Thermo Fisher Scientific) and synthesized by General Biosystems. Either a carboxyrhodamine or Cy5 dye reporter was incorporated at the 5′‐ end of each probe and a nonfluorescent quencher was incorporated at the 3′‐ end. The sensitivity and specificity were evaluated by performing sequence alignments using GenBank data and the Basic Local Alignment Search Tool in NCBI, respectively.

**TABLE 3:** Primers and probes for *Acinetobacter baumannii* and *Klebsiella pneumoniae* detection

Name	Target Gene	Sequence (5′–3′)
Ab‐F	OXA‐51‐like β‐lactamase (*bla_OXA_*_‐_ *_51_*‐*like*)	CAC ACT ACG GGT GTT TTA GTT ATC CA
Ab‐R		CGA GCA AGA TCA TTA CCA TAG CTT T
Ab‐Probe		Cy5‐CAA GGC CAA ACT C‐MGB
Kp‐F	*Klebsiella pneumoniae* hemolysin *(khe)*	GGG CGA GGT TTA CGT CTC AA
Kp‐R		GCG TGT GGA TAA GAG GTG CG
Kp‐Probe		ROX‐CCA CCA CGA GCG GC‐MGB

Abbreviations: Ab, *Acinetobacter baumannii*; F, forward primer; Kp, *Klebsiella pneumoniae*; R, reverse primer.

### 2.5 ddPCR

A duplex ddPCR assay was performed using a Pilot Gene Droplet Digital PCR System (Pilot Gene Technology, Hangzhou, China) to detect *A*. *baumannii* and *K*. *pneumoniae* simultaneously in one chip following the manufacturer's protocol. Briefly, the ddPCR master mix for each testing panel had a final volume of 15 μl comprising 1 × ddPCR premix, 1 μM forward and reverse primers, 300 nM of each probe, and 5 μl of isolated plasma DNA. The mixture was loaded onto a ready‐to‐use disposable plastic chip. Approximately 20,000 droplets were generated using a droplet generator (DG32; Pilot Gene Technology), followed by amplification on a TC1 thermal cycler (Pilot Gene Technology). The thermal cycling parameters were 95°C for 5 min, followed by 40 cycles at 95°C for 15 s and 60°C for 60 s. After PCR amplification, the droplets were analyzed using an iScanner 5 chip scanner (Pilot Gene Technology). Data analysis for droplet counts and amplitudes was performed with 30 min of hands‐on time using GenePMS software version v2.0.01.20011.

### 2.6 Evaluation of specificity, sensitivity, linearity, and precision

Analytical specificity was evaluated by detecting the genomic DNA isolated from the positive and negative control bacterial strains. Analytical sensitivity was determined via limit of blank (LoB) and limit of detection (LoD) assays using 22 replicate samples. Probit analysis was performed to measure the LoD of each bacterium. The mean copy numbers and standard deviations (SDs) were calculated. LoB was calculated as mean_blank_ + 1.645 (SD_blank_). LoD was calculated as mean_blank_ + 3 (SD_blank_) (Armbruster & Pry, 2008). Linearity was determined via two‐fold serial dilution of the DNA template, with each dilution being measured in eight replicates. Precision was evaluated by testing five different concentrations of genomic DNA in eight replicates.

### 2.7 Statistical analysis

Note, SAS 9.13 (SAS Institute) was used for database management and statistical analyses. Continuous variables were expressed as the mean ± SD or median and interquartile range (IQR) where appropriate. The *t*‐test was performed to analyze normally distributed continuous variables, whereas the Mann–Whitney *U* test was performed to analyze non‐normally distributed continuous variables. Categorical variables were reported as frequencies and percentages and were analyzed using the chi‐squared test. Values of *p* < 0.05 were considered significant.

## 3 RESULTS

### 3.1 Analytical specificity

We designed a specific primer‐probe set for each strain to detect *A*. *baumannii* and *K*. *pneumoniae* in the blood samples. The specificity test showed that all 5 *A*. *baumannii* isolates and the 33 *K*. *pneumoniae* isolates from ATCC were detected using the corresponding primer‐probe set, whereas the 131 negative control isolates were not detected using the primer‐probe sets (Table 4). These results suggest that each primer‐probe set is specific to either *A*. *baumannii* or *K*. *pneumoniae*.

**TABLE 4:** Analytical specificity and sensitivity of droplet digital polymerase chain reaction for *Acinetobacter baumannii* and *Klebsiella pneumoniae* detection

Microorganism	Specificity		Sensitivity			
	*A. baumannii*	*K. pneumoniae*	Mean	SD	LoB	LoD
*A. baumannii*	5/5 (100)	0/5 (0)	0.09	0.28	0.55	0.93
*K. pneumoniae*	0/33 (0)	33/33 (100)	0.03	0.08	0.16	0.27
Other 131 isolates	0/131 (0)	0/131 (0)				

Abbreviations: LoB, limit of blank; LoD, limit of detection; SD, standard deviation.

### 3.2 Analytical sensitivity

Analytical sensitivity was measured using the LoB and LoD assays. The LoB of *A*. *baumannii* and *K*. *pneumoniae* were 0.55 copies/µl and 0.15 copies/µl, respectively; whereas the LoD of *A*. *baumannii* and *K*. *pneumonia* were 0.93 copies/µl and 0.27 copies/µl, respectively (Table 4). The LoD was defined as the lowest concentration that yielded positive results in the ddPCR assay.

### 3.3 Analytical repeatability, reproducibility, and linearity

Within‐run precision (repeatability) and within‐laboratory precision (reproducibility) were measured using different concentrations of sheared *A*. *baumannii* and *K*. *pneumoniae* DNA samples. The within‐run coefficients of variation (CVs) of *A*. *baumannii* and *K*. *pneumoniae* were 2.7%–5.3% and 2.3%–9.5%, respectively. The within‐laboratory CVs of *A*. *baumannii* and *K*. *pneumoniae* were 5.3%–7.5% and 7.4%–9.7%, respectively (Tables 5 and 6).

**TABLE 5:** Analytical repeatability of droplet digital polymerase chain reaction for *Acinetobacter baumannii* and *Klebsiella pneumoniae* detection

*A. baumannii*		*K. pneumoniae*	
Concentration (copies/µl)	CV %	Concentration (copies/µl)	CV %
578.4 ± 15.9	2.7	551.5 ± 12.6	2.3
261.2 ± 13.4	5.1	240.4 ± 8.2	3.4
91.9 ± 3.2	3.5	90.5 ± 5.6	6.2
46.2 ± 1.4	2.9	62.0 ± 2.6	4.2
26.1 ± 1.4	5.3	36.1 ± 3.4	9.5

Abbreviation: CV, coefficient of variation.

**TABLE 6:** Analytical reproducibility of droplet digital polymerase chain reaction for *Acinetobacter baumannii* and *Klebsiella pneumoniae* detection

*A. baumannii*		*K. pneumoniae*	
Concentration (copies/µl)	CV %	Concentration (copies/µl)	CV %
580.1 ± 30.6	5.3	541.1 ± 40.0	7.4
91.0 ± 5.5	6.0	91.5 ± 7.0	7.6
27.1 ± 2.0	7.5	38.5 ± 3.7	9.7

Abbreviation: CV, coefficient of variation.

**FIGURE 1:**
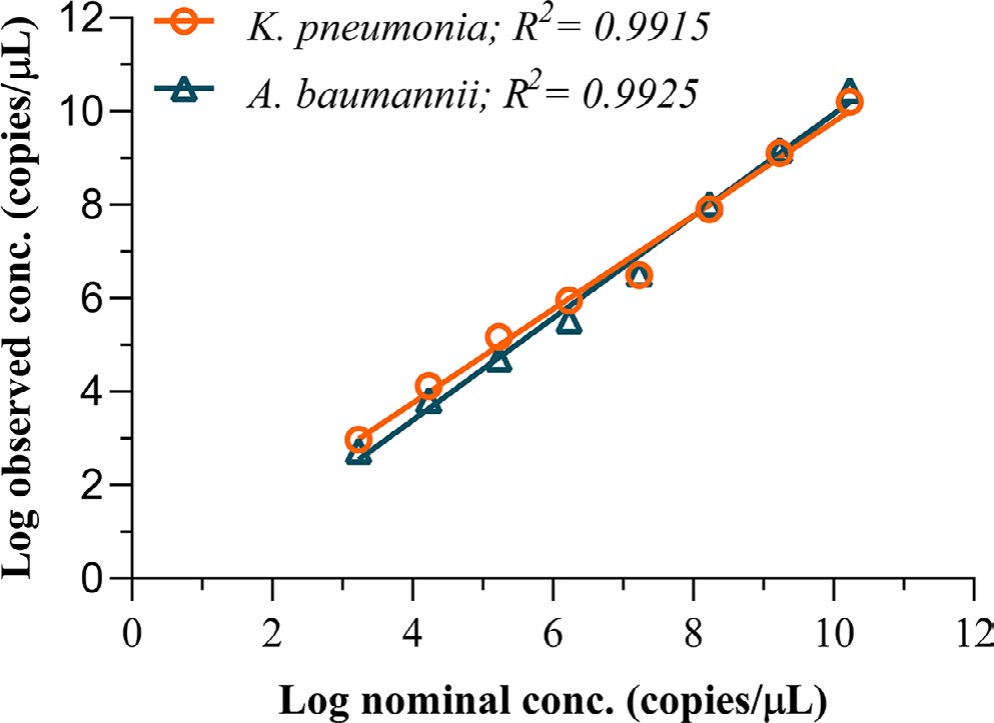
Linear regression analysis. Linearity was determined via two‐fold serial dilution of the DNA template

Linearity was determined using two‐fold serial dilutions of the DNA template. The regression lines representing the linear relationships between DNA copy numbers and concentrations of *A*. *baumannii* (*R* ^2^ = 0.9925) and *K*. *pneumoniae* (*R* ^2^ = 0.9915) are shown in Figure 1. Taken together, these results indicate that ddPCR has excellent repeatability, reproducibility, and linearity in detecting *A*. *baumannii* and *K*. *pneumoniae* in blood samples.

### 3.4 Turnaround time of diagnosis

Blood samples were obtained and transported to the ddPCR laboratory in approximately 10 min. Plasma was immediately isolated after centrifugation for 20 min. DNA extraction and PCR amplification were completed within 3 h. Data analysis was performed within 30 min using the GenePMS software. The average turnaround time for the ddPCR assay was 4.2 ± 0.51 h, which was remarkably shorter than that of the blood culture method (90.6 ± 10.9 h, *p* < 0.01).

### 3.5 Clinical performance

Of the 170 patients with suspected BSIs tested via ddPCR, 53 showed positive results, including 23 (13.5%, 23/170) *A*. *baumannii* cases, 26 (15.3%, 26/170) *K*. *pneumoniae* cases, and 4 (2.4%, 4/170) dual infection cases. In contrast, blood cultures alone detected only 4 (2.4%) *A*. *baumannii* cases, 7 (4.1%) *K*. *pneumoniae* cases, and 22 (12.9%) cases with other bacterial infections, with an overall positivity rate of 19.4% (33/170). All 11 cases (100%, 11/11) of *A*. *baumannii* or *K*. *pneumoniae* infections detected via blood culture showed positive results in the ddPCR assay. The positivity rate of *A*. *baumannii* and *K*. *pneumoniae* determined via the ddPCR assay was much higher than that determined via blood culture (31.2%, 53/170 vs. 6.5%, 11/170) (Table 7).

**TABLE 7:** Clinical validation of ddPCR analysis vs. blood culture

Species	ddPCR	Blood culture
*Acinetobacter baumannii*	23	4
*Klebsiella pneumoniae*	26	7
*A. baumannii* & *K. pneumoniae*	4	0
Other microorganisms	Not detected	22
Negative	117	137

Abbreviation: ddPCR, droplet digital polymerase chain reaction.

The clinical characteristics of the 53 patients that tested positive via ddPCR assays are summarized in Table 3. No significant differences were observed in age, systolic and diastolic blood pressure, plasma C‐reactive protein levels, white blood cell counts, serum creatinine, or the use of vasoactive drugs (all *p* ≥ 0.05) between the patients that tested positive based on ddPCR alone (*n* = 42) and the patients that tested positive via both ddPCR and blood culture (*n* = 11). Compared to 11 patients that were reported positive via both ddPCR and blood culture, the 42 patients reported positive via ddPCR alone had significantly decreased serum concentrations of procalcitonin (3.70 vs. 12.9 pg/L, *p* = 0.03) and lactate (2.76 vs. 4.26 mM, *p* = 0.04), APACHE II scores (21.9 vs. 27.9, *p* = 0.02), SOFA scores (10.5 vs. 13.8, *p* = 0.03), and 28‐day mortality rates (70.6% vs. 90.1%, *p* = 0.04) (Table 3). These data suggest that patients with non‐severe BSIs that may be misdiagnosed via blood culture assays, may still benefit from ddPCR testing and achieve better clinical outcomes.

## 4 DISCUSSION

Blood culture is performed as the gold standard for detecting pathogens in BSIs; however, it has a long turnaround time and relatively low sensitivity. By studying 165,593 blood specimens from 13 hospitals in the USA, Tabak et al. showed that the median time required to identify BSI pathogens using traditional blood culture is 44.0 h, with a sensitivity of approximately 70% for critically ill patients and even lower for fastidious microorganisms (Tabak et al., 2018). To overcome the shortcomings of blood culture in BSI diagnosis, we developed a culture‐independent ddPCR method to rapidly and accurately identify *A*. *baumannii* and *K*. *pneumoniae* in blood samples of patients with suspected BSIs.

Since ddPCR shows higher sensitivity, accuracy, and replication than those of conventional methods, it has a great potential to identify pathogenic microorganisms. In addition to the use of broad‐range primers for the amplification of highly conserved bacterial 16S rRNA and fungal 28S rRNA which enables the detection of BSIs and discrimination between fungal and bacterial infections (Wouters et al., 2020; Ziegler et al., 2019), ddPCR is also widely applied to detect and quantify specific pathogenic microorganisms, such as Hepatitis B virus (HBV) (Huang et al., 2015), *Mycobacterium tuberculosis* (Devonshire et al., 2015; Pan et al., 2021), *Aspergillus terreus* (Alanio et al., 2016), methicillin‐resistant *Staphylococcus aureus* (Luo et al., 2017), and severe acute respiratory syndrome coronavirus 2 (SARS‐CoV‐2) (Liu et al., 2020; Suo et al., 2020). Recently, in the application of SARS‐CoV‐2 nucleic acid detection, ddPCR has been shown to offer greater advantages for the clinical diagnosis of Coronavirus Disease 2019 (COVID‐19) to reduce false‐negative results compared to those obtained via quantitative reverse transcription‐PCR (RT‐qPCR) (Park et al., 2021), although RT‐qPCR is widely used as the gold standard for the clinical detection of SARS‐CoV‐2. In discharged patients with COVID‐19, ddPCR is more suitable for quantification of SARS‐CoV‐2 with a lower viral load compared to using RT‐qPCR, and significantly improves the accuracy of diagnosis of patients with COVID‐19 who relapse (Liu et al., 2020).

Our results showed that ddPCR could identify *A*. *baumannii* and *K*. *pneumoniae* in whole blood samples within 4 h, with a specificity of 100% for each strain and detection limit of 0.93 copies/µl for *A*. *baumannii* and 0.27 copies/µl for *K*. *pneumoniae*. This is consistent with the results of a previous study which reported that ddPCR requires only 0.54 ± 0.94 copies of covalently closed circular DNA for accurate HBV detection (Mu et al., 2015). Clinical validation of 170 patients with suspected BSI showed that ddPCR not only identified patients who tested positive based on blood culture but also detected those who tested negative via blood culture. Notably, compared to patients who tested positive via both blood culture and ddPCR assay, the patients who tested positive via ddPCR alone had less severe clinical symptoms and better clinical outcomes, suggesting that these patients benefited from the treatment recommended based on ddPCR‐based diagnosis in the early stages of infection. Thus, ddPCR may serve as a rapid and reliable method to identify causal pathogens and guide treatment decisions in the early stages of BSI.

The human immune system and antibiotic treatment kill invading pathogens in BSIs, leading to the release of nucleic acids from pathogens into the blood; such nucleic acids are considered part of circulating cfDNA. Thus, the presence of specific pathogenic DNA among cfDNA can reflect the presence of pathogens in the bloodstream. Accumulating evidence has demonstrated the feasibility of NGS of plasma cfDNA to identify pathogens in BSI (Blauwkamp et al., 2019; Grumaz et al., 2019; Rossoff et al., 2019). However, the typical turnaround time of 2 d and the high cost of NGS represent barriers to the application of cfDNA NGS in clinical practice (Crawford et al., 2020; Simner et al., 2018). In this study, we exploited the ultra‐high sensitivity of ddPCR and the feasibility of cfDNA in pathogen identification to develop a ddPCR‐based assay using cfDNA as the template. The turnaround time of ddPCR to diagnosis was estimated as 4 h, which is significantly shorter than that of NGS (2–3 days) (Grumaz et al., 2016) or blood culture (90.6 ± 12.9 h in this study).

Wouters et al. developed a ddPCR method to detect bacteria and fungi using metagenomic DNA as the template and broad‐range primer‐probe sets; however, the overall specificity in clinical validation is only 80% (Wouters et al., 2020). In this study, we used cfDNA as the template and designed specific primer‐probe sets for *A*. *baumannii* and *K*. *pneumoniae*. We achieved 100% specificity for detecting well‐characterized ATCC and CMCC isolates of each species, which was higher than those reported for other PCR‐based methods, such as SeptiFast (50% according to Warhurst et al., 2015, and 85.5% according to Korber et al., 2017), Magicplex (29%; Zboromyrska et al., 2019), TAC assay (79.4%; Zhang et al., 2018), T2Bacteria (90%; Maki, 2019; Nguyen et al., 2019), and cfDNA NGS (93.7%; Blauwkamp et al., 2019). The relatively low specificity in the detection of metagenomic DNA may be partially attributed to the large amount of human DNA that interferes with primer and probe binding during species‐specific PCR assays.

The sensitivity of blood culture assays is typically low; the blood culture‐based positivity rate of patients with sepsis has been reported as 51% over 22 years in the United States (Martin et al., 2003). Cheng et al. reported a 71.7% blood culture‐based positivity rate in patients with severe sepsis from ten university hospitals in China (Cheng et al., 2007). Similarly, 70% of patients with sepsis admitted to the ICU in a 1‐day international investigation were reported to be positive based on blood culture tests (Vincent et al., 2009). The low sensitivity of blood cultures may be attributed to the low abundance of bacteria in the blood, antibiotic treatment before sampling, and culture techniques. Molecular detection methods are less affected by these factors; thus, they usually report higher positivity rates than those obtained via blood cultures. The positivity rates determined via different molecular methods are 1.56 to 6.45‐fold higher than those determined via blood culture (Farnaes et al., 2019; Grumaz et al., 2019; Korber et al., 2017; Long et al., 2016; Nguyen et al., 2019). In the present study, the positivity rates of *A*. *baumannii* and *K*. *pneumoniae* were 6.8‐fold ([23 + 4] / 4) and 4.3‐fold ([26 + 4] / 7) higher than those determined via blood culture, respectively. Thus, molecular detection methods may detect pathogens that are not detected via blood culture, thereby providing timely diagnosis and appropriate antibiotic treatment for patients with BSIs.

In this study, the 53 patients who tested positive via ddPCR assay had typical symptoms resulting from BSIs, including body temperature above 38.5°C, abnormally elevated serum levels of C‐reactive protein, and procalcitonin, hemodynamic instability, and severe organ dysfunction. Notably, the patients who tested positive via ddPCR alone exhibited less severe symptoms than those reported positive via both ddPCR and blood culture, suggesting that ddPCR is more sensitive than blood culture for early diagnosis of BSIs.

This study had certain limitations. First, as the microbial cfDNA is possibly released from dead pathogens and is continuously detected almost 2 weeks after conventional blood cultures report negative results (Eichenberger et al., 2021), the presence of microbial cfDNA in the blood does not necessarily indicate the presence of living microorganisms in the bloodstream and may also be derived from a previous BSI. Second, unlike metagenomic NGS that has a wider coverage for detecting causative pathogens, ddPCR can only identify a small number of target pathogens; thus, it is not possible to determine whether a patient has a mono‐microbial infection or polymicrobial infection based on the detection results from the ddPCR assay alone. In addition, for multiple pathogens detected via ddPCR, it is difficult to determine if a pathogen associated with a relatively low cfDNA load in the blood sample is clinically relevant. Third, it is challenging to distinguish causative pathogens from normal microbes or environmental contaminants. Although the ddPCR assay can report quantitative results, there are no clear cutoffs that differentiate infection from colonization or contaminants. Therefore, to partially overcome the aforementioned limitations, enlargement of assay panels to cover additional pathogens and tracking the microbial cfDNA load change may be helpful, as the dynamic monitoring of microbial cfDNA concentration may reflect the progress of active BSIs. When determining the clinical significance of a pathogen detected via cfDNA sequencing, the entire clinical scenario should be considered.

In conclusion, we developed a novel ddPCR method to detect two major pathogens in patients with suspected BSI. Clinical validation revealed that our method was superior to blood culture in terms of specificity, sensitivity, and turnaround time, and represents a promising method for the early and accurate diagnosis of BSIs. However, in this pilot study, we only evaluated two major Gram‐negative bacteria responsible for BSIs. Other clinically important pathogens should be investigated in future studies.

## CONFLICT OF INTEREST

None declared.

## AUTHOR CONTRIBUTIONS

**Yang Zheng:** Conceptualization (equal); Formal analysis (equal); Writing‐original draft (equal). **Jun Jin:** Conceptualization (equal); Formal analysis (equal); Methodology (equal); Writing‐original draft (equal). **Ziqiang Shao:** Data curation (equal); Methodology (equal); Validation (equal). **Jingquan Liu:** Data curation (equal); Methodology (equal); Project administration (equal). **Run Zhang:** Data curation (supporting); Resources (supporting); Validation (equal). **Renhua Sun:** Investigation (supporting); Supervision (supporting); Writing‐review & editing (equal). **Bangchuan Hu:** Conceptualization (equal); Funding acquisition (equal); Supervision (equal); Writing‐original draft (equal).

## ETHICS STATEMENT

The study protocol was approved by the Institutional Review Board and Ethics Committee of Zhejiang Provincial People's Hospital (No. 2019KY002). Written informed consent was obtained from all patients or their legal representatives.

## Data Availability

All data generated or analyzed during this study are included in this published article.
